# Nutritional and Micronutrient Status of Female Workers in a Garment Factory in Cambodia

**DOI:** 10.3390/nu8110694

**Published:** 2016-11-02

**Authors:** Jan Makurat, Hanna Friedrich, Khov Kuong, Frank T. Wieringa, Chhoun Chamnan, Michael B. Krawinkel

**Affiliations:** 1 Institute of Nutritional Sciences, Justus Liebig University Giessen, Wilhelmstrasse 20, 35392 Giessen, Germany; hanna-friedrich@gmx.net (H.F.); krawinkel@fb09.uni-giessen.de (M.B.K.); 2 Department of Fisheries Post-Harvest Technologies and Quality Control (DFPTQ), Fisheries Administration, Ministry of Agriculture, Forestry and Fisheries (MAFF), 186 Preah Norodom Boulevard, 12000 Phnom Penh, Cambodia; kuong.kh@gmail.com (K.K.); chhounchamnan@gmail.com (C.C.); 3 UMR 204 Nutripass, Institut de Recherche pour le Développement (IRD), IRD/UM/SupAgro, 911 Avenue d’Agropolis, 34394 Montpellier, France; franck.wieringa@ird.fr

**Keywords:** underweight, anemia, micronutrient deficiency, garment factory, Cambodia, iron, vitamin A, vitamin B12, malnutrition, industry

## Abstract

Background: Concerns about the nutritional status of Cambodian garment workers were raised years ago but data are still scarce. The objectives of this study are to examine the nutritional, hemoglobin and micronutrient status of female workers in a garment factory in Phnom Penh, Cambodia, and to assess if body mass index is associated with hemoglobin and/or micronutrient status. Methods: A cross-sectional survey was conducted among 223 female workers (nulliparous, non-pregnant) at a garment factory in Phnom Penh. Anthropometric measurements were performed and blood samples were taken to obtain results on hemoglobin, iron, vitamin A, vitamin B12 and inflammation status (hemoglobinopathies not determined). Bivariate correlations were used to assess associations. Results: Overall, 31.4% of workers were underweight, 26.9% showed anemia, 22.1% showed iron deficiency, while 46.5% had marginal iron stores. No evidence of vitamin A or vitamin B12 deficiency was found. Body mass index was associated with serum ferritin (negative) and serum retinol-binding protein (positive) concentrations, but not strongly. A comparison between underweight and not underweight workers resulted in distinctions for iron deficiency and iron deficiency anemia, with a higher prevalence among not underweight. Conclusions: The prevalence of underweight, anemia and poor iron status was high. Young and nulliparous female garment workers in Cambodia might constitute a group with elevated risk for nutritional deficiencies. Strategies need to be developed for improving their nutritional, micronutrient and health status. The poor iron status seems to contribute to the overall prevalence of anemia. Low hemoglobin and iron deficiency affected both underweight and those not underweight. Despite the fact that body mass index was negatively associated with iron stores, true differences in iron status between underweight and not underweight participants cannot be confirmed.

## 1. Introduction

After a prolonged period of civil war, the first garment factories were set up in Cambodia in the early 1990s by foreign investments. Before that period the country did not develop any modern garment industry [1,2]. Investments were also mainly attracted by the steady supply of labor force at relatively low wage rates [1,2]. Until today, most factories belong to foreign investors and are located in and around the outskirts of Phnom Penh, the capital of Cambodia [1,2]. The majority of them engage in “cut, make and trim” activities and depend on imported fabrics and machinery, as well as on technical and supervisory personnel from abroad [1,2]. In late 2015, there were in total about 700 factories in Cambodia, employing around 643,000 workers [3].

Some 86% of the garment and footwear workers are female, primarily young women from low-income rural households and many of them have a poor school education, which limits their options for working outside of agriculture and factory labor [4,5]. Additional finances made while working in the garment industry are often sent back to support family members, which has a substantial anti-poverty effect [5,6,7]. In 2015 the minimum wage for garment and footwear workers in Cambodia was 128 USD per month [3]. Beside the minimum salary, workers heavily rely on bonuses and overtime work [5,7]. Concerns about the nutritional status of Cambodian garment workers were raised years ago [6]. Malnutrition among workers has become a sensitive topic and has been linked to the mass faintings that are regularly reported from the factories [5]. Nevertheless, the action undertaken by stakeholders in the past was limited and data on the nutritional situation of garment workers are still scarce. It has been concluded that a decent diet, an adequate living standard and savings might be out of reach for this population [5,6,7]. The average daily amount spent by Cambodian garment workers on food (~1.5 USD) was regarded as not enough to ensure adequate dietary intake [5]. The expense on food is a result of extreme budgeting, achieved by the women through eating and living in groups and minimizing costs by bringing food from their hometowns [5,6,7]. Beyond that, thrift measures also involve workers skipping meals [5,6].

In addition to the nutritional status of female garment workers, the prevalence of anemia and micronutrient deficiencies is of importance in the Cambodian context [8]. According to the Cambodian Demographic Health Survey 2014 (CDHS 2014), 45% of women in reproductive age (15–49 years) were anemic [8]. Anemia of nutritional origin is caused by diets that lack sufficient amounts of essential hematopoietic nutrients, such as iron, vitamin A (VitA), vitamin B12 (VitB12) or folic acid, to meet the need for hemoglobin (Hb) and red blood cell synthesis [9,10]. Non-nutrition factors are especially menstrual blood loss, genetically determined hemoglobinopathies and parasite infestation, such as in malaria and helminths [9,11]. Malnutrition among women in reproductive age, with respect to underweight, anemia and micronutrient deficiencies, is associated with numerous poor health related outcomes, such as impaired cognition, reduced work capacity and impaired immune responses, leading to lowered resistance to infections [9,12,13]. During pregnancy, it is also associated with increased maternal morbidity and mortality, low birth weight, premature delivery and increased fetal and neonatal deaths [9,12,13].

All relevant actors along the Cambodian garment industry should be empowered to make informed choices through better information. However, despite an increasing number of nutrition-related research projects in Cambodia within the last years, there is still poor knowledge on the nutritional situation of female garment workers and just a few reports touched upon this topic [5,14]. The current paper is based on baseline data from the LUPROGAR study (Lunch Provision in Garment Factories), a factory-based randomized controlled trial, whose primary goal is to determine the impact of daily lunch provision through a canteen during workdays on the nutritional status (anthropometry and micronutrient status) among female garment workers in Cambodia. The two objectives of the present survey are to examine the nutritional, Hb and micronutrient status of female workers employed by a garment factory in Phnom Penh, Cambodia, and to assess if body mass index (BMI) is associated with Hb and/or micronutrient status among study subjects.

## 2. Materials and Methods

### 2.1. Study Site and Study Design

In this cross-sectional survey, baseline data from the LUPROGAR study were used. LUPROGAR was implemented at Apsara Garment Co. Ltd., Phnom Penh, Cambodia, an export-oriented garment factory located in the suburban commune Chom Chau in Cambodia’s capital Phnom Penh, approximately 10 km west of the city center. Baseline data were collected in April 2015. The factory was previously not operating a canteen, but a canteen was installed specifically for the LUPROGAR study. Enrolled study participants received either free lunch provision through the newly-established canteen during workdays (intervention arm) or an equal monetary compensation at the end of the study (control arm). At the time of study implementation, the factory employed about 1300 workers, the majority of which were young unmarried women from low-income rural households. Conditions of employment were assumed to be comparable with overall working conditions in the Cambodian garment sector. The factory operated on six workdays per week and was selected purposely, since the management was showing interest to collaborate in this research study.

### 2.2. Sample Size

The LUPROGAR study used an explorative approach (two tail) to estimate an appropriate sample size, as data on the nutritional and micronutrient status of Cambodian garment workers are rare, as well as data on the effects of lunch provision in this context. Calculations were carried out using G*Power software (Version 3.1.9.2, University of Kiel, Kiel, Germany). Assuming a 5% level of significance (alpha = 0.05) and a statistical power of 80% (beta = 0.20) to detect a small to medium standardized effect size of 0.35 (Cohen’s d) between both arms using a two-sided test [15], 130 subjects in each group were required. To allow for about 20% loss to follow up, it was initially aimed at recruiting a total of 330 subjects, 165 subjects in each arm.

### 2.3. Enrolment of Study Participants

Firstly, the factory management, superintendents and union representatives were informed in detail about the objectives and procedure of the LUPROGAR study. Subsequently, the study was announced during a meeting to all factory employees. Written informed consents, including a study description in lay language, were obtained (signature or fingerprint) at lunch breaks and after end of work, prior to any data collection, by trained local project assistants. Workers who signed the informed consent were invited to the baseline assessment, which took place in a separate room during working hours and included a clinical screening performed by trained local nurses. Inclusion criteria for the LUPROGAR study were: female, nulliparous, non-pregnant and <31 years at date of enrolment. The exclusion criteria were: acute or chronic disease requiring treatment and/or medication, handicaps interfering with nutritional and/or health status, blood Hb < 7.0 g/dL, clinical signs of VitA or iodine deficiency and employment as supervisor/superintendent. Workers excluded from participation due to any health issues were referred for treatment.

### 2.4. Questionnaires

Trained local project assistants applied a semi-structured questionnaire, collecting data on background information and the socio-economic status of study participants and their respective households. Obtained data on age of workers was cross-checked with reference data from the factory’s personnel department. Trained local project nurses administered a semi-structured health questionnaire, collecting data on present intake of medications, as well as on illness history and sick leave in the 14 days preceding the interview. Both questionnaires were similar to those from the CDHS 2014 [8] and were pre-tested under field conditions.

### 2.5. Anthropometric Measurements

Weight, height and mid-upper arm circumference (MUAC) of participants were assessed by two trained examiners following the guidelines from the Centers for Disease Control and Prevention (CDC) [16]. Weight was measured without shoes in light clothing to the nearest 0.1 kg, using an electronic SECA-UNICEF scale (UNISCALE, UNICEF supply). Height was measured to the nearest 0.1 cm, using a SECA 213 stadiometer (SECA, Hamburg, Germany). MUAC was measured to the nearest 0.1 cm, using a non-stretchable fiberglass measuring tape to determine the mid-point of the upper arm, and a MUAC measuring tape for adults supplied by UNICEF/WFP. All measurements were taken twice and the mean was used for further analysis. The maximum tolerated differences were 0.5 kg for weight, 1.0 cm for height and 0.5 cm for MUAC, otherwise the measurement was repeated. BMI was calculated and subjects were classified using following cut-off points [16]: severe underweight (BMI < 16.0 kg/m^2^), moderate underweight (BMI 16.0–16.99 kg/m^2^), mild underweight (BMI 17.0–18.49 kg/m^2^), normal weight (BMI 18.5–24.99 kg/m^2^) and overweight (BMI 25.0–29.99 kg/m^2^). All devices and measurement procedures were pre-tested under field conditions.

### 2.6. Blood Sample Collection and Analysis

Samples of 5 mL non-fasting venous blood (venepuncture at left or right arm) were taken by trained local nurses in a separate private area. Immediately after blood was collected, blood drops were put on a hydrophobic glass slide for subsequent twofold blood Hb measurement using a HemoCue Hb 301 photometer (HemoCue AB, Ängelholm, Sweden). Blood left in the syringe was filled into a serum vacutainer with clot activator (Becton Dickinson, Franklin Lakes, NJ, USA) and kept at room temperature for a minimum duration of 1 h to allow for blood clotting and afterwards kept chilled at 4 °C. Then it was separated within 3 h by centrifugation (2700 rpm, calculated equivalent at 1300× *g*, 10 min), aliquoted into capped Eppendorf tubes and again kept chilled at 4 °C. Samples were then transported in a cool box containing ice packs to the Department of Fisheries Post-Harvest Technologies and Quality Control (Phnom Penh, Cambodia) on a daily basis and kept frozen at −25 °C until further processing.

Subsamples for the determination of VitB12 concentration were transported in a cool box containing ice packs to the Pasteur Institute Cambodia (Phnom Penh, Cambodia). Serum VitB12 was measured by electrochemiluminescence (ECL), using a COBAS e 411 immunoassay analyser (Roche Diagnostics, Rotkreuz, Switzerland) with kits and control samples provided by the manufacturer. VitB12 deficiency was defined as serum VitB12 < 148 pmol/L and a marginal VitB12 deficiency as serum VitB12 ≥ 148 and < 222 pmol/L [17]. Remaining serum aliquots were shipped on dry ice to the Institute of Nutritional Sciences at the Justus Liebig University (Giessen, Germany) and stored at −25 °C until they were transported in a cool box containing ice packs to the VitMin laboratory (Willstaett, Germany) for determination of ferritin (FER), soluble transferrin receptor (sTfR), retinol-binding protein (RBP), C-reactive protein (CRP), and α1-acid-glycoprotein (AGP) concentrations. FER, RBP, sTfR, CRP, and AGP, were determined by a sandwich enzyme-linked immunosorbent assay (ELISA) technique [18], using pooled samples for quality control and certified samples (CDC, Atlanta, US and Bio-Rad, Hercules, CA, USA) to establish calibration curves for each indicator. All values represent the mean of an independent double measurement.

Subclinical inflammation was defined as increased CRP (>5 mg/L) and/or increased AGP concentrations (>1 g/L) and categorized into three stages: incubation (high CRP and normal AGP), early convalescence (both CRP and AGP elevated) and late convalescence (high AGP only) [19]. FER concentration was adjusted for inflammation by correction factors for each inflammation stage [19]. Iron deficiency was defined by depleted iron stores (adjusted serum FER < 15 μg/L) [9], tissue iron deficiency by high serum sTfR (>8.3 mg/L) [20] and marginal iron stores by adjusted serum FER ≥ 15 and < 50 μg/L [21]. Serum RBP concentrations were used as a surrogate measure for circulating retinol to evaluate VitA status [22]. RBP values were likewise adjusted for the presence of inflammation by correction factors for each stage of inflammation [23]. VitA deficiency was defined by adjusted serum RBP < 0.70 μmol/L and marginal VitA deficiency by adjusted serum RBP values ≥ 0.70 and < 1.05 μmol/L [22,24].

### 2.7. Data Management and Statistical Analysis

Data entry and validation by double entry of questionnaires and anthropometry sheets was performed by trained project assistants using EpiData software (Version 3.1, EpiData Association, Odense, Denmark). Data management and statistical analyses were executed using SPSS software (Version 22.0.0.1, IBM Corp., Armonk, NY, USA). Normality of distributions was evaluated using the Shapiro-Wilk test. As most continuous variables (background characteristics, anthropometry and micronutrient status) were skewed, descriptive statistics for continuous variables are therefore represented by the median and interquartile range (IQR). Categorical variables are expressed as frequency and percentage. To assess if BMI is associated with Hb and micronutrient status, bivariate correlations between BMI and Hb, serum FER, serum sTfR, serum RBP and serum VitB12 concentrations were calculated with non-parametric Spearman’s correlation. On the basis of illustration purposes, values for serum FER and serum sTfR were log-transformed in the correlation diagrams. The significance was set at 5% (*p*-value < 0.05).

### 2.8. Ethics

The LUPROGAR study was approved by the Institutional Review Board of the Faculty of Medicine at Justus Liebig University, Giessen, Germany (14 November 2014) and the National Ethics Committee for Health Research (NECHR) at the Ministry of Health, Phnom Penh, Cambodia (29 December 2014). Written informed consent was collected from all study participants prior to enrolment by signature or fingerprint. The ethics committees approved the consent format prior to data collection. The study was registered at the German Clinical Trials Register (9 January 2015, Identifier: DRKS00007666).

## 3. Results

### 3.1. Participant Characteristics

A total of 267 female workers signed the informed consent prior to enrolment, of whom 229 were present and 38 were not present (*n* = 30, ceased to work; *n* = 8, refused to participate) at the enrolment procedure. Another six workers were excluded from participation at the clinical screening (*n* = 2, blood Hb < 7.0 g/dL; *n* = 2, not nulliparous; *n* = 1, physical handicap, *n* = 1, chronic disease). Descriptive characteristics of the 223 enrolled study participants based on the questionnaire results are shown in Table 1 and Table 2. Median age of participants was 20.9 years (IQR: 19.3–22.3 years). Median duration of employment in the factory was 10.7 months (IQR: 5.2–20.1 months). 66.4% (*n* = 148) reported a previous employment in another garment factory. Median monthly basic salary among workers was 128.0 USD (IQR: 128.0–133.0 USD), which increased by some 48% with bonus, overtime, and allowance, to a median value for last total monthly salary of 190.0 USD (IQR: 175.0–210.0). Almost all participants (99.6%, *n* = 222) stated regular monthly payments to their family households. The median for this substantial expense was 100.0 USD (IQR: 100.0–150.0 USD), which accounts for approximately 53% of median last total monthly salary.

The vast majority of participants were single (91.9%, *n* = 205). Overall, 39.9% (*n* = 88) did not complete more than a primary school education, with 21.1% (*n* = 47) of workers having left primary school without graduation. 26.5% (*n* = 59) completed secondary school or had a higher schooling. The hometown province of 95.1% (*n* = 212) of study participants was not Phnom Penh. Of the women, 30.5% (*n* = 68) were commuting, travelling between factory and family households in their hometown, while 69.5% (*n* = 155) reported a nearby accommodation on workdays. 65.5% (*n* = 146) stayed in a nearby shared room for rent. The main job types were sewing (63.2%, *n* = 141) and quality control (16.1%, *n* = 36). Their households’ primary sources of income were primarily wage employment and farming with 58.7% (*n* = 131) and 23.8% (*n* = 53), respectively.

The prevalence of self-reported sicknesses and sick leave for a period of 14 days preceding the interview are shown in Table 3, with 45.7% (*n* = 102) of participants reporting a respiratory tract infection, 30.9% (*n* = 69) reporting fever and 20.2% (*n* = 45) reporting diarrhea. Overall, 61.4% (*n* = 137) reported any of these three sicknesses. In contrast to this, only 14.4% (*n* = 32) of workers took sick leave in the same period.

### 3.2. Nutritional Status

The results of the anthropometric assessments among participants are shown in Table 4. Median weight and height were 45.4 kg (IQR: 42.5–49.9 kg) and 153.5 cm (IQR: 150.0–156.9 cm), respectively. BMI values ranged from 14.7 kg/m^2^ (severe underweight) to 27.8 kg/m^2^ (overweight) and the median was 19.6 kg/m^2^ (IQR: 18.3–21.2 kg/m^2^). While 65.9% (*n* = 147) had a normal BMI (18.5–24.99 kg/m^2^), 31.4% (*n* = 70) had a BMI lower than 18.5 kg/m^2^, indicating underweight. Most of the underweight workers fell into the category of mild underweight (BMI 17.0–18.49 kg/m^2^), in total 23.3% (*n* = 52) of all participants. The prevalence of moderate (BMI 16.0–16.99 kg/m^2^) and severe underweight (BMI < 16.0 kg/m^2^) was 5.8% (*n* = 13) and 2.2% (*n* = 5), respectively. Overweight (BMI 25.0–29.99 kg/m^2^) was observed in only 2.7% (*n* = 6).

### 3.3. Hemoglobin and Micronutrient Status

Blood samples were available from 219 participants (*n* = 4, refused blood sampling). Results of the analyses are shown in Table 5. Median Hb was 12.5 g/dL (IQR: 11.9–13.2 g/dL). 26.9% (*n* = 59) of study participants had anemia (Hb < 12.0 g/dL), whereby most of anemic subjects showed a mild anemia (Hb 11.0–11.9 g/dL). The adjusted median value of serum FER was 33.1 µg/L (IQR: 16.9–60.7 µg/L) and 22.1% (*n* = 48) of participants showed iron deficiency (adjusted FER < 15 µg/L), while 46.5% (*n* = 101) had marginal iron stores (adjusted FER ≥ 15 and <50 µg/L). The prevalence of tissue iron deficiency (sTfR > 8.3 mg/L) was lower with 10.1% (*n* = 22). Iron deficiency anemia (Hb < 12.0 g/dL and adjusted FER < 15 µg/L) was found in 12.9% (*n* = 28). Adjusted median serum RBP was 1.38 µmol/L (IQR: 1.21–1.58 µmol/L). None of the participants showed VitA deficiency (RBP < 0.70 µmol/L), while 7.4% (*n* = 16) showed marginal deficiency (RBP ≥ 0.70 and < 1.05 µmol/L). Median serum VitB12 concentration was 400 pmol/L (IQR: 299–513 pmol/L) and only one women showed VitB12 deficiency (VitB12 < 148 pmol/L), while 5.6% (*n* = 12) had a marginal VitB12 deficiency (VitB12 ≥ 148 and < 222 pmol/L).

**Figure 1:**
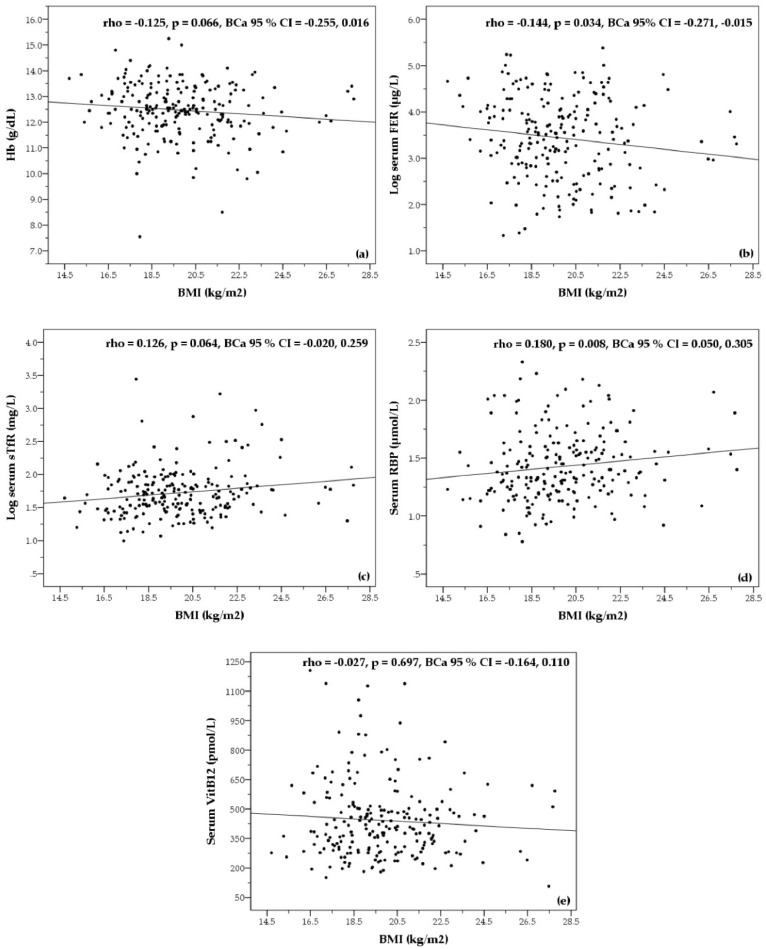
Bivariate correlations between BMI and Hb (**a**); serum FER (**b**); serum sTfR (**c**); serum RBP (**d**); serum VitB12 (**e**) concentrations of female workers employed by a garment factory in Phnom Penh, Cambodia. Correlation coefficients (rho), calculated with non-parametric Spearman’s correlation, include bias corrected and accelerated bootstrap 95% confidence intervals (BCa 95% CI). For illustration purposes, values for serum FER and serum sTfR were log-transformed in the diagrams. Hb: Hemoglobin; FER: Ferritin; sTfR: Soluble transferrin receptor; RBP: Retinol binding protein; VitB12: Vitamin B12; BMI: Body mass index.

Bivariate correlations between BMI and blood Hb concentration, and between BMI and biochemical parameters of micronutrient status are shown in Figure 1. BMI showed only small-sized correlations with Hb, serum FER, serum sTfR and serum RBP concentrations. BMI was negatively correlated with serum FER (rho = −0.144, *p* = 0.034, BCa 95% CI = −0.271, −0.015) and positively correlated with serum RBP (rho = 0.180, *p* = 0.008, BCa 95% CI = 0.050, 0.305). However, the relationships between BMI and Hb (rho = −0.125, *p* = 0.066, BCa 95% CI = −0.255, 0.016) and between BMI and serum sTFR (rho = 0.126, *p* = 0.064, BCa 95% CI = −0.020, 0.259) were not significant. No relationship between BMI and serum VitB12 was found (rho = −0.027; *p* = 0.697, BCa 95% CI = −0.164, 0.110).

Prevalence of anemia, micronutrient deficiencies and subclinical inflammation for underweight (BMI < 18.5 kg/m^2^) and not underweight participants (BMI ≥ 18.5 kg/m^2^) are shown in Table 6. Distinctions were observed for iron deficiency (adjusted FER < 15 µg/L) and iron deficiency anemia (Hb < 12.0 g/dL and adjusted FER < 15 µg/L), with higher prevalence among not underweight workers. 5.9% (*n* = 4) of underweight workers showed iron deficiency anemia, while the prevalence among not underweight workers was 16.1% (*n* = 24). The prevalence of iron deficiency among workers with a BMI ≥ 18.5 kg/m^2^ was 26.2% (*n* = 39), compared to underweight workers with 13.2% (*n* = 9). No relevant differences between groups were found for anemia (Hb < 12.0 g/dL), marginal iron stores (adjusted FER ≥ 15 and < 50 µg/L), tissue iron deficiency (sTfR > 8.3 mg/L), VitA deficiencies (RBP < 0.70 µmol/L and RBP ≥ 0.70 and < 1.05 µmol/L), VitB12 deficiencies (VitB12 < 148 pmol/L and VitB12 ≥ 148 and < 222 pmol/L) and subclinical inflammation (CRP > 5.0 mg/L and/or AGP > 1.0 g/L).

## 4. Discussion

In this paper, it is shown that the nutritional status of female garment workers in Cambodia might be of concern, with underweight (BMI < 18.5 kg/m^2^), anemia (Hb < 12.0 g/dL) and iron deficiency (serum FER < 15 µg/L) being prevalent among study participants.

Underweight was found in approximately one-third (31.4%) of the women. Although most underweight workers showed mild underweight (BMI 17.0–18.49 kg/m^2^), the term “mild” in this classification should not veil the various serious consequences of it [12,13]. According to the World Health Organization (WHO), a prevalence of 20%–39% underweight in a given population is considered a critical situation [12]. A similar prevalence of underweight (36%) among female garment workers in Cambodia has been reported by NGOs in 2013, based on a small cross-sectional survey [5]. On the contrary, a recent International Labour Organization (ILO) study conducted in several Cambodian factories found a distinctly lower prevalence of 14.3% underweight among female workers, who were mainly married, not nulliparous and whose age was therefore higher [14]. The subjects enrolled in this study were relatively young, nulliparous (inclusion criteria) and mainly single. They might constitute a group of workers who is especially at risk for underweight, with a prevalence rate that is considerably higher than the national estimate for underweight of women of reproductive age (15–49 years) in Cambodia (which is 14.0%) [8]. Many young workers will likely start their employment when they are already underweight, as shown for adolescent female garment workers in Bangladesh [25]. Underweight among Cambodian women is especially widespread among young women aged 15–19 years (27.5%) [8].

It is presumed that expenses on food, and hence dietary intake in terms of quantity and quality, might be compromised by the limited financial means of study participants. Disposable income of workers is mainly determined by remittances, i.e., regular monthly payments to their family households. These financial commitments were the largest expense among workers in this study (on average 53% of total monthly salary). Expenses on food among Cambodian garment workers increased within the last years, as consumer prices did as well, but have been continuously described as low and insufficient to ensure an adequate dietary intake [5,7]. However, reliable data on the actual dietary intake among Cambodian garment workers are missing and further research with respect to this aspect should be undertaken.

Infectious diseases are known to have negative effects on nutritional status, and vice versa; a poor nutritional status interferes with immune functions and thereby enhances the risk for infections [26]. Study participants frequently reported symptoms of respiratory tract infections (45.7%), fever (30.9%) and diarrhea (20.2%) in the 14 days preceding the interview, with 61.4% who reported at least one of these. However, no differences in the prevalence of sicknesses and sick leave among underweight and not underweight study participants were observed (data not shown). In addition, only 14.4% of subjects stated that they have taken sick leave in the same period, leading to the conclusion that many workers tend to continue work despite being sick.

Anemia affected approximately one out of four subjects (26.9%). According to the WHO, anemia is a public health problem when the prevalence is >20% [9]. The prevalence of anemia among participants was expected to be higher, since the CDHS 2014 found that 45% of Cambodian women of reproductive age were anemic [8]. The same high prevalence among female garment workers was reported by the recent ILO survey [14]. Similar to the data presented here, rates of 30% anemia among non-pregnant Cambodian women have been reported [27]. Currently, the contributors to the high prevalence of anemia in Cambodia are still not fully understood [28]. In the past, iron deficiency was regarded as the most important factor, however, recent studies have shown a low prevalence of iron deficiency and concluded a low impact on hemoglobin concentrations among Cambodian women [24,28]. Genetic hemoglobin disorders lead to lower hemoglobin concentrations and an increased risk of anemia [27]. According to the literature, these inherited hemoglobinopathies affect >50% of the Cambodian population, the most common include hemoglobin E variants and α-thalassemia, resulting in reduced or abnormal hemoglobin synthesis [27,29,30]. Although the prevalence of hemoglobinopathies was not determined in the study population, it is likely that these disorders contribute to the prevalence of anemia. Recently, it was questioned if increasing the provision of iron will improve anemia in the Cambodian setting [28]. Measures to reduce zinc and folic acid deficiency, as well as to treat and prevent hookworm infections, were suggested to be included in current interventions [28].

In the current study, approximately one-fifth (22.1%) of the participants were iron deficient and approximately one-half (46.5%) of the women showed marginal iron store values and will be especially at risk to become iron deficient if pregnancy occurs [21]. These figures are higher compared to representative prevalence rates for Cambodian women (iron deficiency <10%; marginal iron stores ~40%) [24,28]. In addition, 10.1% showed tissue iron deficiency. The prevalence of iron deficiency anemia (simultaneous low Hb and iron deficiency) among women in the present study was 12.9%. Considering this, poor iron status partially explains the prevalence of anemia in this population. Animal source foods are a primary source for dietary iron intake [31]. However, in Cambodia they belong to the most expensive food products [32]. Even though recent findings suggest that iron-rich foods (e.g., flesh meat, fish) are daily consumed by a majority of female garment workers [14], the quantities might be too small to meet dietary reference intakes (DRI). Data supporting this assumption were reported for Cambodian women in a rural area [32].

No evidence of VitA (0.0%) or VitB12 deficiency (0.5%) was found among subjects and the prevalence of marginal deficiencies for both were <10% (7.4% marginal VitA deficiency; 5.6% marginal VitB12 deficiency), which is in line with national representative data [24,28]. It is concluded, that the VitA and the VitB12 status of participants is not of concern and is not likely contributing significantly to the anemia burden in the studied women [9].

The secondary objective of this study was to examine associations between BMI and hemoglobin as well as micronutrient status among the participating women. In a simple bivariate correlation analysis, BMI showed only small-sized associations with serum FER and RBP. Small-sized effects between BMI and Hb, and BMI with serum sTfR were not significant and no association between BMI and serum VitB12 concentration was observed.

Opposite to the expectations, BMI was negatively associated with serum FER (rho = −0.144, *p* = 0.034, BCa 95% CI = −0.271, −0.015). A comparison between underweight and not underweight workers resulted in distinctions for iron deficiency and iron deficiency anemia, with a 2–2.5 times higher prevalence among participants with a BMI > 18.5 kg/m^2^. Still, it is to be noted that the relationship between BMI and serum FER was only marginally significant and that the number of cases with iron deficiency and iron deficiency anemia within both groups were relatively small. Therefore, true differences in iron status between underweight and not underweight participants cannot be confirmed. Amenorrhea (absence of menstruation) is known to be linked with underweight [33] and, although no information on menstrual blood loss was obtained in this study, could be considered regarding a slightly better iron status in underweight workers. But, the inverse relationship between BMI and iron status has been consistently reported by others, especially in overweight/obese compared to normal weight individuals [34]. On the contrary, a cross-sectional study among 1530 Vietnamese women of reproductive age reported positive associations between BMI with Hb and plasma FER, although this was not associated with a different prevalence of anemia or iron deficiency among different BMI groups [35].

BMI was positively associated with serum RBP (rho = 0.180, *p* = 0.008, BCa 95% CI = 0.050, 0.305), but this weak relationship did not result in a distinctly different prevalence of marginal VitA deficiency between underweight and not underweight subjects. Low plasma retinol concentrations and a higher prevalence of marginal VitA status among underweight women were reported from the mentioned study in Vietnam [35]. The authors showed that food energy intake among participants increased along the BMI classifications and concluded that higher micronutrient intakes could have resulted in a better VitA status of individuals with a BMI > 18.5 kg/m^2^. No data on energy or micronutrient intake among participants were collected in the present study, however, this conclusion could be also valid for the study population. Moreover, the uptake of VitA and carotenoids is linked to dietary fat intake [36], which in turn is positively associated with BMI.

### Limitations of the Study

A main limitation of the present study was the monocentric cross-sectional design, in which findings cannot be derived beyond the locality and population included in this survey. Study participants could not get randomly selected and this survey was conducted with a relatively small number of women. However, it is assumed that this does not restrict the interpretation of the results obtained, since inclusion criteria represented the majority of garment workers employed by the factory. Initially, it was planned to enroll 330 participants for the LUPROGAR study. Fear and reservations related to the blood sampling procedure were reported by many workers, especially due to headlines about a HIV outbreak caused by unlicensed clinicians reusing syringes shortly before the baseline assessment [37].

The prevalence of hemoglobinopathies was not measured, although this is likely to be a contributing factor to the observed prevalence of anemia. Furthermore, menstrual blood loss, a determinant of iron stores in women of reproductive age [38], was not recorded. Inherited hemoglobin disorders are also known to impact on iron markers [27,39]. Recently, these effects were examined in a cross-sectional survey among 450 Cambodian women [39]. In this study, only the Hb EE genotype (found in ~7% of subjects) was significantly associated with a 50% higher geometric mean ferritin concentration, compared to those with normal Hb AA genotype. The prevalence of iron deficiency (ferritin < 15 µg/L) did not differ between subjects with any abnormal Hb genotype and subjects with Hb AA. Regarding sTfR values, the authors reported that Hb EE and Hb Constant Spring genotypes (the later affecting ~4% of subjects) were significantly associated with a 51% and 44% increase in geometric mean sTfR concentration, respectively. Consequently, the prevalence of tissue iron deficiency (sTfR > 8.3 mg/L) among women with any hemoglobinopathy was significantly higher than among women with normal Hb AA (~26% vs. ~10%). The prevalence of elevated sTfR was especially high (55%) among Hb EE subjects. Based on their findings, the authors conclude that ferritin values, in contrast to sTfR, appear to reflect more accurately iron deficiency in Cambodian women [39]. In the current study, main statements regarding the iron status of participants are based on the ferritin data, which remain informative, although ferritin values might not represent true iron status among a subgroup with Hb EE disorder. sTfR concentration as an indicator of iron status seems to be less reliable in the Cambodian context [39].

Moreover, RBP is a proxy indicator for retinol concentrations and although the correlation between retinol and RBP is high, as retinol is bound to RBP in a 1:1 ratio, RBP concentrations tend to underestimate VitA deficiency due to the presence of holo-RBP (RBP without retinol) in the circulation, which is especially the case at lower retinol concentrations [40]. However, as the prevalence of RBP < 1.05 µmol/L is also low (<10%), it is assumed that our finding on frank VitA deficiency in this population is valid.

## 5. Conclusions

The prevalence of underweight, anemia and poor iron status among study participants is of concern. According to the present study, approximately two-thirds of subjects (iron deficient or with marginal iron stores) will have a high risk for iron deficiency when they get pregnant. Young and nulliparous garment workers in Cambodia are identified as a part of the workforce with a high risk for undernutrition and an elevated risk for nutritional deficiencies. Therefore, strategies need to be developed for improving their nutritional, micronutrient and health status. In addition, adequate national actions to tackle malnutrition among female adolescents should be undertaken. Despite a recent rise in minimum salaries, as seen for the Cambodian garment industry within the last years, higher salaries might not automatically lead to improved nutrition among workers in the short term, as they primarily fulfil a major contribution to the social securing of their family households. Consequently, improving national social and health security systems, especially for low-income rural households, might be one pathway that could directly lead to increased disposable incomes and indirectly lead to improved nutrition among garment workers.

In contrast to recent nationwide results among women of reproductive age, the poor iron status in the study population seems to contribute to the overall prevalence of anemia. However, the VitA and VitB12 status of participants is not of concern. Low hemoglobin and iron deficiency affected both groups, those underweight as well as those normal and overweight, unexpectedly with a higher prevalence among participants with a BMI > 18.5 kg/m^2^. Despite the fact that BMI was negatively associated with iron stores, true differences in iron status between underweight and not underweight participants cannot be confirmed. As reported in other studies, it should be noted that hemoglobinopathies (not determined in this study) are likely to impact on Hb concentration (decreased Hb) and markers of iron status (partly increased values of FER and sTFR).

The overall findings should have practical implications for the design and implementation of programs and strategies aiming at the improvement of the nutritional and micronutrient status among young female garment workers in Cambodia.

## Figures and Tables

**Table 1:** Descriptive characteristics (continuous variables) of female workers employed by a garment factory in Phnom Penh, Cambodia ^1^.

Characteristics	Median	IQR
General		
Age (years)	20.9	19.3–22.3
School attendance (years)	7.0	6.0–9.0
Duration of employment (months)	10.7	5.2–20.1
Income		
Monthly basic salary (USD)	128.0	128.0–133.0
Last monthly salary, incl. bonus, overtime, and allowance (USD) ^2^	190.0	175.0–210.0
Expense		
Monthly payment to family household (USD) ^3^	100.0	100.0–150.0
Household		
Number of people in household	5.0	4.0–6.0

^1^ Total *n* = 223; ^2^ *n* = 215 (*n* = 8, newcomer (≤1 month of employment) without previous monthly salary); ^3^ *n* = 222 (*n* = 1, worker without monthly payment to family household); IQR: Interquartile range; USD: United States Dollar.

**Table 2:** Descriptive characteristics (categorical variables) of female workers employed by a garment factory in Phnom Penh, Cambodia ^1^.

Characteristics	*n*	%
Marital status		
Single	205	91.9
Married	14	6.3
Widowed/divorced	4	1.8
Religion		
Buddhist	223	100.0
Level of education		
Some primary	47	21.1
Completed primary (grade 6)	41	18.4
Some secondary	76	34.1
Completed secondary (grade 9)	41	18.4
Some high school	10	4.5
Completed high school (grade 12)	8	3.6
Hometown province		
Phnom Penh	11	4.9
Others	212	95.1
Accommodation on workdays		
Hometown, family household	68	30.5
Nearby place of friend/family	7	3.1
Nearby shared room for rent	146	65.5
Nearby private room for rent	2	0.9
Job type in factory		
Sewing	141	63.2
Quality control	36	16.1
Buttoning	15	6.7
Cutting	9	4.0
Packaging	9	4.0
Assistant	5	2.2
Others	8	3.6
Previous employment in other garment factory		
Yes	148	66.4
No	75	33.6
Households primary source of income		
Wage employment	131	58.7
Farming	53	23.8
Casual labor	17	7.6
Business/petty trade	13	5.8
Others	9	4.0

^1^ Total *n* = 223.

**Table 3:** Self-reported sickness and sick leave in the 14 days preceding the interview among female workers employed by a garment factory in Phnom Penh, Cambodia ^1^.

Variables	*n*	%
Self-reported sickness		
Respiratory tract infection	102	45.7
Fever	69	30.9
Diarrhea	45	20.2
Any of these sicknesses	137	61.4
Sick leave taken	32	14.4

^1^ Total *n* = 223.

**Table 4:** Anthropometry and nutritional status of female workers employed by a garment factory in Phnom Penh, Cambodia ^1^.

Variables	Median or *n*	IQR or %	Min.	Max.
Anthropometry				
Weight (kg)	45.4	42.5–49.9	34.9	68.2
Height (cm)	153.5	150.0–156.9	142.5	166.6
MUAC (cm)	23.7	22.2–25.4	19.0	33.0
BMI (kg/m^2^)	19.6	18.3–21.2	14.7	27.8
Nutritional status				
Underweight (BMI < 18.5 kg/m^2^)	70	31.4		
Mild (BMI 17.0–18.49 kg/m^2^)	52	23.3		
Moderate (BMI 16.0–16.99 kg/m^2^)	13	5.8		
Severe (BMI < 16.0 kg/m^2^)	5	2.2		
Normal (BMI 18.5–24.99 kg/m^2^)	147	65.9		
Overweight (BMI 25.0–29.99 kg/m^2^)	6	2.7		

^1^ Total *n* = 223; IQR: Interquartile range; Min.: Minimum; Max.: Maximum; MUAC: Mid upper-arm circumference; BMI: Body mass index.

**Table 5:** Hemoglobin, iron, vitamin A, vitamin B12 and subclinical inflammation status of female workers employed by a garment factory in Phnom Penh, Cambodia.

Variables	Median or *n*	IQR or %	Min.	Max.
Hemoglobin status ^1^				
Hb (g/dL)	12.5	11.9–13.2	7.6	15.3
Anemia (Hb < 12.0 g/dL)	59	26.9		
Mild (Hb 11.0–11.9 g/dL)	42	19.2		
Moderate (Hb 8.0–10.9 g/dL)	16	7.3		
Severe (Hb < 8.0 g/dL)	1	0.5		
Iron status ^2^				
Serum FER, unadjusted (µg/L)	34.5	17.6–62.4	3.8	217.8
Serum FER, adjusted ^3^ (µg/L)	33.1	16.9–60.7	3.8	217.8
Serum sTfR (mg/L)	5.4	4.4–6.5	2.7	31.2
Deficiency (adjusted ^3^ serum FER < 15 µg/L)	48	22.1		
Marginal stores (adjusted ^3^ serum FER ≥ 15 and < 50 µg/L)	101	46.5		
Tissue iron deficiency (serum sTfR > 8.3 mg/L)	22	10.1		
Iron deficiency anemia ^2^				
Hb < 12.0 g/dL and adjusted ^3^ serum FER < 15 µg/L	28	12.9		
Vitamin A status ^2^				
Serum RBP, unadjusted (µmol/L)	1.37	1.21–1.57	0.70	2.33
Serum RBP, adjusted ^3^ (µmol/L)	1.38	1.21–1.58	0.78	2.33
Deficiency (adjusted ^3^ serum RBP < 0.70 µmol/L)	0	0.0		
Marginal deficiency (adjusted ^3^ serum RBP ≥0.70 and < 1.05 µmol/L)	16	7.4		
Vitamin B12 status ^4^				
Serum VitB12 (pmol/L)	400	299–513	107	1206
Deficiency (serum VitB12 < 148 pmol/L)	1	0.5		
Marginal deficiency (serum VitB12 ≥ 148 and < 222 pmol/L)	12	5.6		
Subclinical inflammation ^2^				
CRP (mg/L)	0.23	0.13–0.48	0.02	43.55
AGP (g/L)	0.58	0.48–0.72	0.26	2.50
Incubation (CRP > 5 mg/L only)	2	0.9		
Early convalescence (AGP > 1 g/L and CRP > 5 mg/L)	4	1.8		
Late convalescence (AGP > 1 g/L only)	14	6.5		

^1^ Total *n* = 219; ^2^ Total *n* = 217 (*n* = 2, no aliquot); ^3^ Values adjusted for inflammation as described in methods section; ^4^ Total *n* = 216 (*n* = 3, no aliquot); IQR: Interquartile range; Min.: Minimum; Max.: Maximum; Hb: Hemoglobin; FER: Ferritin; sTfR: Soluble transferrin receptor; RBP: Retinol binding protein; CRP: C-reactive protein; AGP: α1-acid-glycoprotein.

**Table 6:** Anemia, micronutrient deficiencies and subclinical inflammation by underweight and not underweight female workers employed by a garment factory in Phnom Penh, Cambodia.

Variables	BMI < 18.5 (kg/m^2^)		BMI ≥ 18.5 (kg/m^2^)	
	*n*	%	*n*	%
Anemia ^1^				
Hb < 12.0 g/dL	17	24.6	42	28.0
Iron deficiency anaemia ^2^				
Hb < 12.0 g/dL and adjusted ^3^ serum FER < 15 µg/L	4	5.9	24	16.1
Iron ^2^				
Deficiency (adjusted ^3^ serum FER < 15 µg/L)	9	13.2	39	26.2
Marginal stores (adjusted ^3^ serum FER ≥ 15 and < 50 µg/L)	32	47.1	69	46.3
Tissue iron deficiency (serum sTfR > 8.3 mg/L)	6	8.8	16	10.7
Vitamin A ^2^				
Deficiency (adjusted ^3^ serum RBP < 0.70 µmol/L)	0	0.0	0	0.0
Marginal deficiency (adjusted ^3^ serum RBP ≥ 0.70 and < 1.05 µmol/L)	7	10.3	9	6.0
Vitamin B12 ^4^				
Deficiency (serum VitB12 < 148 pmol/L)	0	0.0	1	0.7
Marginal deficiency (serum VitB12 ≥ 148 and < 222 pmol/L)	5	7.5	7	4.7
Subclinical inflammation ^2^				
Any inflammation phase (CRP > 5.0 mg/L and/or AGP > 1.0 g/L)	4	5.9	16	10.7

^1^ Total *n* = 219 (*n* = 69, BMI < 18.5 kg/m^2^; *n* = 150, BMI ≥ 18.5 kg/m^2^); ^2^ Total *n* = 217 (*n* = 68, BMI < 18.5 kg/m^2^; *n* = 149, BMI ≥ 18.5 kg/m^2^); *n* = 2, no aliquot; ^3^ Values adjusted for inflammation as described in methods section; ^4^ Total *n* = 216 (*n* = 67, BMI < 18.5 kg/m^2^; *n* = 149, BMI ≥ 18.5 kg/m^2^); *n* = 3, no aliquot; BMI: Body mass index; Hb: Hemoglobin; FER: Ferritin; sTfR: Soluble transferrin receptor; RBP: Retinol binding protein; VitB12: Vitamin B12; CRP: C-reactive protein; AGP: α1-acid-glycoprotein.

## Abbreviations

The following abbreviations are used in this manuscript:

USD	United States Dollar
CDHS	Cambodia Demographic Health Survey
VitA	Vitamin A
VitB12	Vitamin B12
Hb	Hemoglobin
LUPROGAR	Lunch Provision in Garment Factories
BMI	Body mass index
UNICEF	United Nations Children’s Emergency Fund
MUAC	Mid upper-arm circumference
FER	Ferritin
sTfR	Soluble transferrin receptor
RBP	Retinol binding protein
CRP	C-reactive protein
AGP	α1-acid-glycoprotein
IQR	Interquartile range
Min.	Minimum
Max.	Maximum
WHO	World Health Organization
NGO	Non-government organization
ILO	International Labour Organization
HIV	Human immunodeficiency virus
